# Early-life glucocorticoids programme behaviour and metabolism in adulthood in zebrafish

**DOI:** 10.1530/JOE-15-0376

**Published:** 2016-07-01

**Authors:** K S Wilson, C S Tucker, E A S Al-Dujaili, M C Holmes, P W F Hadoke, C J Kenyon, M A Denvir

**Affiliations:** The University/BHF Centre for Cardiovascular ScienceUniversity of Edinburgh, The Queen’s Medical Research Institute, Edinburgh, UK

**Keywords:** glucocorticoids, zebrafish, heart, embryo, adult

## Abstract

Glucocorticoids (GCs) *in utero* influence embryonic development with consequent programmed effects on adult physiology and pathophysiology and altered susceptibility to cardiovascular disease. However, in viviparous species, studies of these processes are compromised by secondary maternal influences. The zebrafish, being fertilised externally, avoids this problem and has been used here to investigate the effects of transient alterations in GC activity during early development. Embryonic fish were treated either with dexamethasone (a synthetic GC), an antisense GC receptor (GR) morpholino (GR Mo), or hypoxia for the first 120h post fertilisation (hpf); responses were measured during embryonic treatment or later, post treatment, in adults. All treatments reduced cortisol levels in embryonic fish to similar levels. However, morpholino- and hypoxia-treated embryos showed delayed physical development (slower hatching and straightening of head–trunk angle, shorter body length), less locomotor activity, reduced tactile responses and anxiogenic activity. In contrast, dexamethasone-treated embryos showed advanced development and thigmotaxis but no change in locomotor activity or tactile responses. Gene expression changes were consistent with increased (dexamethasone) and decreased (hypoxia, GR Mo) GC activity. In adults, stressed cortisol values were increased with dexamethasone and decreased by GR Mo and hypoxia pre-treatments. Other responses were similarly differentially affected. In three separate tests of behaviour, dexamethasone-programmed fish appeared ‘bolder’ than matched controls, whereas Mo and hypoxia pre-treated fish were unaffected or more reserved. Similarly, the dexamethasone group but not the Mo or hypoxia groups were heavier, longer and had a greater girth than controls. Hyperglycaemia and expression of GC responsive gene (*pepck*) were also increased in the dexamethasone group. We conclude that GC activity controls many aspects of early-life growth and development in the zebrafish and that, like other species, manipulating GC status pharmacologically, physiologically or genetically in early life leads to programmable metabolic and behavioural traits in adulthood.

## Introduction

In humans, high circulating GC levels are associated with low birth weight, which leads to increased risk of cardiovascular disease, and altered behaviour in adulthood (McCabe *et al*. 2001, Seckl & Meaney 2004, Tegethoff *et al*. 2009, Braun *et al*. 2013, Reynolds 2013). This is commonly referred to as early-life programming and has been widely studied in rodents where maternal GC treatment has been shown to increase blood pressure (Levitt *et al*. 1996, Tang *et al*. 2011), impair glucose intolerance (Nyirenda *et al*. 1998), reduce exploratory behaviour (Welberg *et al*. 2000) and impair memory (Welberg et al. 2000, Kamphuis *et al*. 2003, 2004) in adulthood. Similar traits have been observed in offspring when placental metabolism of endogenous GC is inhibited. However, these studies, which generally involve maternal treatment with GC, do not consider foetal autonomy, the placental barrier or the interrelationship between foetal and maternal endocrine systems. For example, foetal GC levels are determined partly by *de novo* foetal steroidogenesis and partly by transfer from the maternal circulation. Maternal treatment with low-dose dexamethasone could, therefore, cause foetal GC levels to either increase or decrease depending on rates of placental transfer of synthetic and endogenous GC hormones. The extent to which endogenous GC production by the foetal and maternal hypothalamus-pituitary-adrenal (HPA) axes may also be affected.

The zebrafish has been exploited as a readily accessible, transparent animal model for investigating cardiovascular disease. As a vertebrate, zebrafish have the equivalent endocrine systems as mammals (Spitsbergen & Kent 2003, McGonnell & Fowkes 2006). While they do not synthesise aldosterone (the main mammalian mineralocorticoid), the zebrafish hypothalamus-pituitary-interrenal (HPI) axis is analogous to the mammalian HPA axis (Alsop & Vijayan 2009, Aluru & Vijayan 2009, Lohr & Hammerschmidt 2011, Tokarz *et al*. 2013*a*). Similar to mammals, fish corticosteroids control many key processes linked to the immune system (Chatzopoulou *et al*. 2015), metabolism (Mommsen *et al*. 1999), cognition and behaviour (Clark *et al*. 2011, Griffiths *et al*. 2012, Stewart & Kalueff 2012) in addition to piscine-specific roles in maintaining water and electrolyte homeostasis in an aqueous environment (McCormick 2011, Kwong & Perry 2013). As a non-viviparous species, zebrafish offer a way to explore the impact of diminished maternal influences on GC action during early life. External fertilisation allows early development to be manipulated, directly by injection or by changing environmental conditions, from the egg stage.

While recent publications have highlighted the developmental aspects of altered embryonic glucocorticoid exposure in zebrafish (Hillegass *et al*. 2008, Pikulkaew *et al*. 2011, De Marco *et al*. 2013, Nesan & Vijayan 2013, 2016, Faught *et al*. 2016), few longitudinal studies have been carried out to establish whether embryonic glucocorticoid exposure affects adult phenotypes. In this study, we utilised three protocols to physiologically, pharmacologically and genetically alter GC exposure during the first 120 h post fertilisation (hpf). These protocols have been informed by previous findings indicating that the glucocorticoid receptor (GR) is expressed from the egg stage onwards and evidence of a shift in the source of endogenous GC hormone from a residual maternal supply in the yolk sac to independent *de novo* synthesis in free-swimming 5-day-old larvae (Pikulkaew *et al*. 2010, Wilson *et al*. 2013). The three protocols included: (i) limiting the effects of GC from the earliest stage by injecting eggs with an antisense GR morpholino (GR Mo); (ii) increasing GC activity by adding dexamethasone, a synthetic GC hormone, to the water; and (iii) using a hypoxic environment to evoke a stress response mimicking stagnant water conditions that might occur in nature.

The experimental outcomes of the treatments were compared at two stages. First, to confirm that treatments were effective, various assessments were made at embryonic and larval stages to assess metabolism, development, and behaviour and HPA activity. Secondly, to establish whether early treatments in embryonic fish programme responses in adulthood, assessment of parameters reflecting behaviour, endocrine responses and metabolism were measured in adult zebrafish 84 days after each of the early-life treatment protocol.

## Materials and methods

### Ethical approval

All experiments were carried out in accordance with the accepted standards of humane animal care under the regulation of the Animal (scientific procedures) Act UK 1986 and were approved by the University of Edinburgh Animal Ethics Committee.

### Zebrafish care and husbandry

Adult wild-type fish were housed according to standard operating procedures (Westerfield 2000, Brand *et al*. 2002) and maintained with a 14h light:10h darkness photoperiod cycle at an ambient temperature of 28.5°C. Adult zebrafish were housed in 10L tanks at a density of 2–3 fish per litre (maximum of 30 fish per tank). Containers of marbles were placed in tanks before onset of the light cycle to encourage spawning. Collected eggs were stored in ‘systems water’ containing 0.03% salt (Tropic Marin, Wartenberg, Germany) and 0.5 mg/L of the antiseptic methylthioninium chloride (methylene blue) in deionized water. All fertilised eggs were transferred at the 2–8 cell stage, ~1hpf; to 10cm Petri dishes at 28.5°C with systems water replaced every 24h.

### Embryonic manipulation

All treatments were initiated at the 2–8 cell stage and continued until 120hpf. To increase GC activity, larvae were bathed continuously in normal system water containing the synthetic GR agonist dexamethasone dissolved in ethanol to give a final concentration of 100µM. Control fish were bathed in systems water containing vehicle (0.1 % ethanol). Solutions were replaced every 48 h. Pilot experiments based on published data (Cole *et al*. 2012) with various concentrations of ethanol during the first 120hpf of development established that ≤0.1% ethanol had no significant effects on survival or structural phenotype score (Supplementary Fig. 1, see section on supplementary data given at the end of this article). A recent study by Baiamonte and coworkers (2015) also found no effect of ethanol on embryo morphology but noted a diminished transient acute response in whole-body cortisol to air stress. Although, theoretically, ethanol exposure could be a contributory factor in the current dexamethasone experiments, it should be noted that controls for these experiments were treated with similar concentrations of ethanol. Also, results from controls of the dexamethasone group were not different from the controls in the hypoxia and GR morpholino group which were not exposed to ethanol.

Previous work involving a dose-ranging study (1–200μM) established 100µM dexamethasone as the optimum concentration of dexamethasone in the bathing water that affected physiology (Wilson *et al*. 2013) without impacting on survival of larvae to 120hpf (dexamethasone 87.3±0.9% vs vehicle controls 93.00±2.1%, *P*=0.06). This concentration, 100µM, is greater than that required to suppress endogenous HPI activity (Liu 2007, To *et al*. 2007) but less than that shown to have gross morphological effects (Hillegass *et al*. 2008, Wilson *et al*. 2013). It has also been used by several other groups (Elo *et al*. 2007, Tsuji *et al*. 2014, Chatzopoulou *et al*. 2015).

The global expression of endogenous GR was reduced in larvae by microinjection at the two cell stage with an antisense morpholino (Mo) targeted towards the zebrafish GR gene (GeneTools, Philomath, OR, USA). Two sequences were injected: a translational blocking Mo (GR Mo-cattctccagtcctccttgatccat) and a 5 base pair mispair Mo (mism/control Mo-cattgtccactcctgcttcatcgat) used as an injection control. Both forms of Mo had a 3′ modification to incorporate a red-emitting fluorescent tag (Lissamine) that demonstrated the extent of integration in the developing embryo. Microinjection was standard (Nusslein-Volhard & Dahm 2002) using an IM-300 Narishige programmable microinjector. Injection volume was calculated by measuring the droplet radius (4/3πr^3^). The concentration of morpholino injected (3ng/nL) was titrated to produce a consistent knockdown of zebrafish Gr protein without causing non-specific developmental abnormalities. Previous work in our lab has suggested that the impact of GR Mo on reducing GR protein expression lasts for approximately 5–7 days (Wilson *et al*. 2015).

The effects of hypoxia (5% O_2_) during early development were studied in groups of 20 larvae held from the 2-cell stage for up to 120hpf in a hypoxic chamber (Coy cabinet). Larvae were maintained in Petri dishes with systems water at 28°C in a gaseous environment of 5% O_2_, 5% CO_2_ and 90% N_2_ and a relative humidity of 45%. Controls were maintained under normoxic conditions in otherwise identical conditions.

The impact of each of these three interventions on structural development of larvae was assessed using a phenotype scoring system (Supplementary Fig. 1).

### Embryo/larval developmental assessments

#### Hatch rate, embryogenesis body length and growth rate

Spontaneous chorion hatching occurs between 48 and 72hpf. Embryos and larvae were monitored at 24h intervals over 120hpf to determine whether manipulation increased or decreased the rate of hatching. The total number of larvae hatched was expressed as a percentage of the surviving population.

Body length was determined *post hoc* by image analysis of larvae which had been manually dechorionated using fine forceps. All images were captured using a Leica MZ16F dissecting microscope and camera (DFC320FX) and analysed using ImageJ software. For each length measurement, the time post fertilisation was also recorded allowing growth rate to be determined as the hourly change in length (μm/h).

During normal development, the angle between the middle of the head (a line connecting the eye and ear) and the notochord progressively increases up to around 120hpf (Kimmel *et al*. 1995); this angle is generally referred to as the head–trunk angle. As a measure of change in global development, this was assessed using images of developing larvae acquired at 24, 48 and 72hpf in each of the three treatment groups.

#### Histology

Histological examination was carried out as described previously (Sabaliauskas *et al*. 2006). Briefly, larvae were given anaesthetic overdose (MS-222), fixed in 4% paraformaldehyde (PFA) and embedded in 2% agarose then subsequently paraffin embedded. These paraffin blocks were sectioned into 5 µM thin sections and stained with haematoxylin and eosin (H&E) using a standard protocol (Fischer *et al*. 2008).

### Larval behavioural assessments

#### Larval movement

At 48hpf, larval movements are in the form of random twitches rather than coordinated locomotor activity (Selderslaghs *et al*. 2013). The influence of pharmacological or genetic manipulation on this phenomenon was determined by recording video images of 20 larvae from each of the three treatment groups (and controls) maintained in 5cm Petri dishes over a 5min time period using a digital CCTV camera (Baxall, AD group, Warrington, Cheshire, UK) at ×10 magnification. The number of twitches per minute for each larva in a cohort of 20 was documented using slow-speed playback.

At 96hpf, coordinated muscular movement is usually observed in response to touch stimulus. This reflex was tested by touching larvae gently with Dumont #5 forceps; fish that swam to evade the stimulus were scored 1, while no detectable movement was scored 0. A mean percentage score for each treatment group was calculated from responses of 10 larvae in 5 separate experiments (i.e. a total of 50 larvae/treatment group).

#### Larval swim activity

Locomotor (swim) activity was assessed at 120hpf by open-field observation. Briefly, a single larva was introduced into the mid-region of a 5cm Petri dish marked with three concentric rings, defining inner, mid and outer regions as described by Levin & Cerutti (2009). Video images documenting larvae swim movements were acquired from above the dish using a digital CCTV camera (Baxall, AD group, UK). Data were stored digitally and analysed offline with software (Limelight Ltd, Sutton on Trent, Nottinghamshire, UK) designed to determine total distance travelled, percentage of time spent in each region of the Petri dish and average speed of movement for each larvae *N*=3 (4 embryos per replicate) per group.

### Embryo to adult programming study

The impact of early-life manipulations were then studied in adult zebrafish (84 days post fertilisation-dpf) derived from each of the three treatment groups. Fish were maintained under normal husbandry conditions after cessation of early stage treatments. Age-matched adult controls were derived from vehicle-treated larvae for the dexamethasone group, larvae injected with a mismatch GR Mo and larvae maintained at standard normoxic conditions as part of the hypoxia experimental protocol.

### Developmental and morphometric assessments in adults

#### Condition factor

Adult fish were weighed in a 500mL beaker; the average weight of individual fish was calculated from triplicate measurements. Body length, from head to tail (not including tail fin rays), of anaesthetized fish placed laterally on a moistened sponge was determined using digital callipers (Digital Electronic Vernier Calliper, Sealey, Colchester, Essex, UK). The condition factor (K) (Siccardi *et al*. 2009) was calculated from the equation:


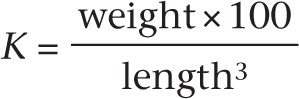


#### Blood glucose

Blood glucose concentration of samples collected from decapitated fish was determined using a Freestyle Freedom Lite blood glucose monitoring system (a system compliant with a small sample volume). Briefly, adult fish were killed by anaesthetic overdose (4.2% MS-222) and blood was collected by decapitation and exsanguination. A minimum of 0.3µL whole blood was placed onto blood glucose monitoring strips as per manufacturer’s instructions. Due to small blood volume and fast coagulation, blood glucose values were averaged from only two measurements/fish. Eight adult fish were assessed per group to provide an overall group mean.

#### Free swimming

After 120hpf (5dpf) in 10cm Petri dishes, larval zebrafish were transferred to 5L breeding tank containing 1L of systems water (unflowing); here, larval fish were observed until 20dpf when they were transferred to adult 10L holding tanks as per husbandry requirements. From 5dpf until 20dpf, the percentage of fish observed to be ‘free swimming’ was assessed. This involved counting the number of larvae moving around the tank. Data are measurements of a minimum of five tanks, observed three times on a given day.

### Adult behavioural assessments

#### Forced dive swim assay

Using an adaption of previously published protocols (Egan *et al*. 2009, Levin & Cerutti 2009, Cachat *et al*. 2010, Rosemberg *et al*. 2011), adult zebrafish were placed individually into a ¾ full, 1.5L trapezoidal tank (15.2 height×27.9 top×22.5 bottom×7.1 widthcm) illuminated from above using a standard angle poise desk lamp and from below by a light box (X-Ray MG-7 Standard 43×36cm Illuminator Light Box, Wolf). Generally, more anxious fish spend longer at the bottom of a novel tank and bolder fish spend more time at the top. A CCTV camera was mounted to provide a side view of the tank. Time spent in the upper and lower regions of the tank, the number of crossings between regions, the total distance travelled and swim velocity over a 5min period were analysed from digital video recordings using Limelight software. Data was assessed in 12 adult fish per treatment group and of respective controls.

#### Open-field assay

Previously published protocols (Egan *et al*. 2009, Levin & Cerutti 2009, Stewart *et al*. 2012) were used for open-field testing. Briefly, a single fish was introduced into a square tank (15×15cm) containing systems water to a depth of 3cm. The tank was illuminated from below with a light box, and from above by a desk lamp. A CCTV camera suspended above the tank recorded video images. Behaviour was analysed for sequential 5min video recordings using Limelight software. The software delineated a 3×3 square grid arena and calculated the total distance, number of grid crossings, time spent in each area and the average velocity accordingly. Once again, 12 adult fish from each of the treatment groups were assayed along with their respective controls.

#### Novel object assay

The novel object assay used the same tank and camera set up as for the open-field assay. A novel object (aquarium enrichment ornament, Pets at Home, UK) was placed in the centre of the tank to represent a predator. The tank was divided into a central region (predation area) containing the novel object (plus a 2cm border) and the rest of the tank (avoidance area). The time spent in either the predation or the avoidance area was recorded and analysed over a 5min period using Limelight software (Limelight Ltd, UK). Bolder fish spent more time in the predation region (Levin & Cerutti 2009). For the study, 12 adult fish from each of the treatment groups along with their respective controls were assayed.

### Whole embryo cortisol

As described previously (Wilson *et al*. 2013), larvae were killed (4.2% MS-222) over ice and rinsed well in systems water to remove any residual drug or methylene blue. Ten larvae were pooled and homogenized in 5mL ice-cold aqueous methanol (1:4 (v/v) systems water:methanol). Homogenates were incubated at 4°C for a minimum of 48h then briefly sonicated and centrifuged at 7000***g*** for 15min at 4°C. Two millilitre aliquots of supernatant were evaporated to dryness under nitrogen. Cortisol in extracts was measured by in-house enzyme-linked immunosorbent assay (ELISA) as published previously (Wilson *et al*. 2013).

### Adult swim water cortisol

Cortisol was assessed in the bathing water of free-swimming adult zebrafish by solid-phase extraction of steroids followed by ELISA. Similar protocols have been used by others (Scott & Ellis 2007, Félix *et al*. 2013). Briefly, five fish were placed in a 1L tank. For each pre-treatment (dexamethasone, GR Mo, hypoxia and their respective controls), three groups of five fish were assigned as basal (unstressed) controls and three groups of five were assigned as the stressed groups. Stressed groups and basal groups were kept in the same room under the same environmental conditions but were separated to limit possible visual stress. Tanks were not directly aerated; however, daily water exchange was carried out with aerated water (oxygen level of 6mg/L). Cortisol was measured from exchanged water following a net/handling stress protocol, which has been shown to increase cortisol levels (Ramsay *et al*. 2009). The experimental protocol began with a 24h baseline familiarisation period, followed by a 24h stress period (repeated netting every 30min for 5h).

Steroids from 1L of holding water were extracted using Sep-Pak®Classic C18 Cartridges (Waters, Hertfordshire, UK) as per manufacturer’s instructions. Steroids were eluted from the cartridge into glass tubes with 2mL methanol and stored at −20°C. Pilot studies with radioactively labelled steroid showed that 100% of steroid was recovered from 1L of water in this way. The methanol eluent was evaporated to dryness under a stream of nitrogen and cortisol was measured by ELISA (Wilson *et al*. 2013).

### Gene expression analysis

Total RNA was extracted from pools of 10 larvae or 5 adult organs using RNeasy Mini kits (Qiagen) according to manufacturer’s instructions. Genomic DNA was removed using a DNA-free Kit (Applied Biosystems). RNA quantity was determined by measuring absorbance at 260nm using a Nano-drop spectrophotometer ND-1000 (Fisher Scientific). RNA integrity was indicated by a 260/280nm absorbance ratio of ~2 and by agarose gel electrophoresis. Total RNA was reverse transcribed using a high-capacity cDNA Reverse Transcription Kit (Applied Biosystems), according to manufacturer’s guidelines. A number of genes were investigated; *gr,* 11β hydroxylase (*cyp11c1* an orthologue of the mammalian Cyp11b1 gene), 11β hydroxysteroid dehydrogenase 2 (*hsd11β2*), insulin-like growth factor 1 (*igf*), FK506 binding protein 5 (*fkbp5*) and mineralocorticoid receptor (*mr*) mRNA levels were measured by quantitative real-time polymerase chain reaction (qRT-PCR, primer sequences supplementary Table 1) using the LightCycler 480 system and universal probes library (UPL) probes (both Roche Diagnostics). Standard operating conditions were 95°C for 5min, 50 cycles (95°C 10s, 60°C 30s, 72°C 1s), 40°C 30s. For each experiment, housekeeping genes (elongation factor 1 alpha (*ef1α*) and glyceraldehyde 3-phosphate dehydrogenase (*gapdh*) were quantified concurrently with genes of interest. Results were calculated with the LightCycler software provided by manufacturer using the maximum second derivative method which considers the entire amplification curve not just the threshold point.

### GR morpholino rescue

To determine whether the effects observed in GR Mo-treated larvae were due specifically to Gr knockdown or to non-specific effects, a rescue experiment was carried out using a modified 5′-UTR zebrafish *gr* mRNA (rescue). As the rescue has a modified 5′UTR, it contains no target for the ATG Mo, but the coding region of the rescue contains the sequence to encode the protein of interest. Template DNA was obtained in the form of IMAGE clone (Source Bioscience, Cambridge, UK), in purified plasmid Miniprep form. The *gr* gene was cloned into the pNR-LIB clone (Source Bioscience, Cambridge, UK) and linearised using XhoI restriction enzyme (Applied Biosystems). Capped transcription reactions were carried out using the mMessage mMachine Kit (Ambion) according to the manufacturer’s instruction. Final capped mRNA concentrations were achieved by dilution with sterile RNAse-free dH_2_O.

### Statistics

Data are presented as mean±s.e.m., unless otherwise stated. Statistical analysis was carried using GraphPad Prism 5.0. One-way or two-way repeated measures analysis of variance (ANOVA) followed by Tukey’s *post hoc* tests were used to compare means within and between groups. Hatch rate was analysed by chi-squared analysis of proportions by calculating the degree of difference between the observed data and the null hypothesis (based on the control data). Sex ratios were analysed by Fisher’s test as the degree of difference between observed data and control data. Statistical significance throughout was accepted as *P*<0.05 (*P≤0.05, **P ≤ 0.01, ***P ≤ 0.001).

## Results

### Larval growth and development

#### Hatch rate

**Figure 1:**
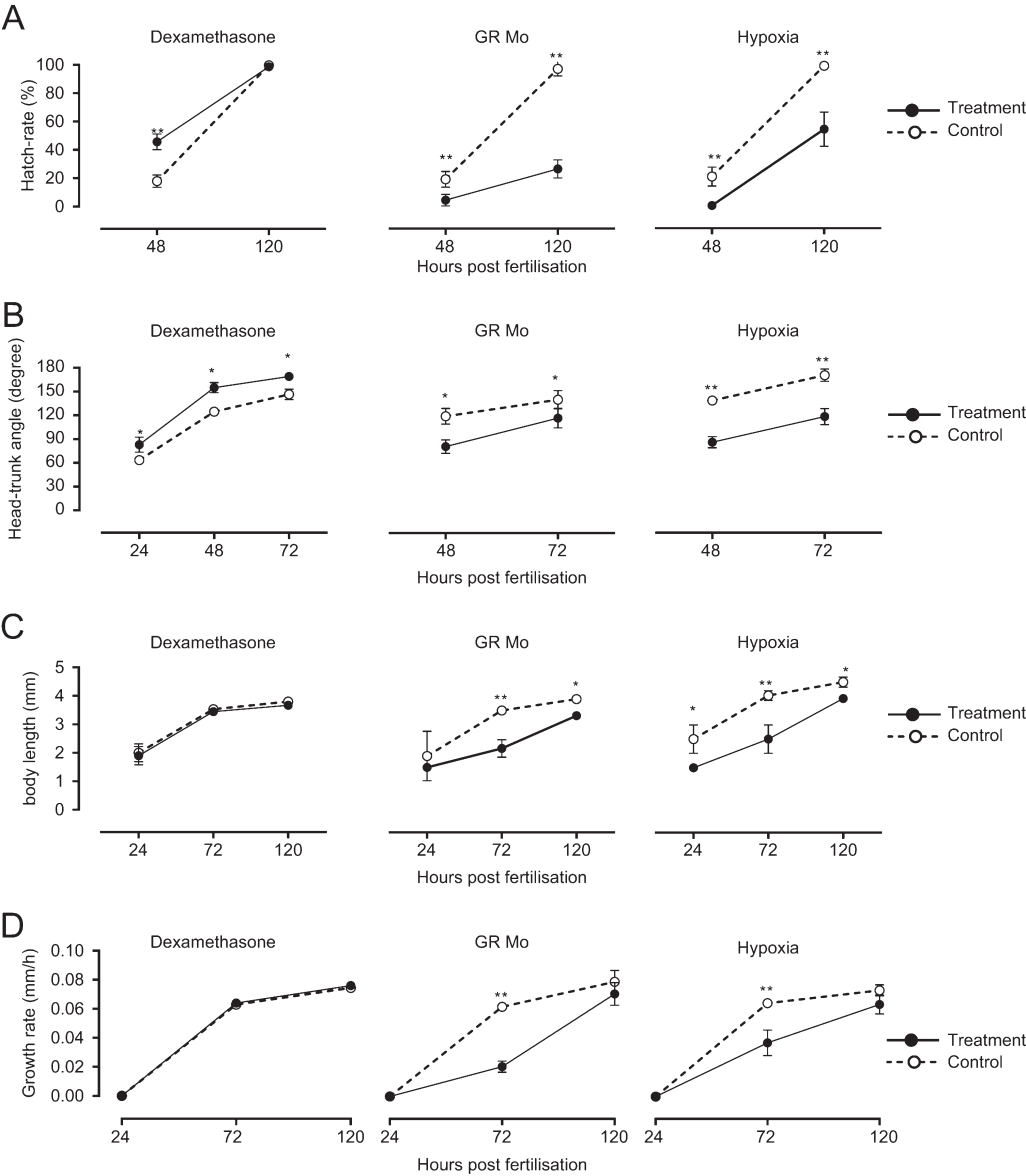
Effects of embryonic treatment on larval growth and development. Results show the effects of dexamethasone, glucocorticoid receptor (GR) morpholino knockdown (GR Mo) and hypoxia treatment during 120h post fertilisation (hpf) on (A) chorion hatch rate, (B) head–trunk angle, (C) body length and (D) growth rate. For each treatment (●), results are compared with respective controls (○, vehicle, mismatched GR Mo or normoxia) by one-way ANOVA and Bonferroni *post hoc* analysis *P≤0.05, **P≤0.001. Results are mean±s.e.m. from *n*=3 experiments with 20 larvae/group for each experiment).

Dexamethasone treatment more than doubled the percentage of hatched larvae at 48hpf, whereas hypoxia and GR Mo treatments significantly reduced the numbers of hatched larvae compared with controls at 48hpf and remained at less than half the number of controls by 120hpf (Fig. 1A).

#### Head–trunk angle

Head–trunk angle (HTA), a measure of global development of the head and axial skeleton, was advanced by dexamethasone treatment and delayed by hypoxia and GR Mo treatment (Fig. 1B). HTA was significantly greater in dexamethasone-treated fish at each of the stages analysed. These differences in HTA correspond to a 6–8h delay in development in GR Mo and hypoxic fish and 8h advancement in development following treatment with dexamethasone (Kimmel *et al*. 1995). Rescue of GR Mo, using mRNA, partially restored HTA to normal values (Supplementary Fig. 2).

#### Growth

Total body length was reduced by hypoxia and GR Mo treatment and was unaffected by dexamethasone (Fig. 1C). The effects of hypoxia on body length were consistently less than controls throughout the treatment period (*P*<0.01). In the GR Mo group, body length was initially lower but recovered to control levels by 120hpf. Rate of growth was fastest in the dexamethasone group in the first 24h, slowed over the next 48h and slowed further over the subsequent 48 h. In contrast, growth rate was slowed by hypoxia and GR Mo, but increased steadily between 24 and 120hpf (21–24 µm/h for Mo and 13–14µm/h with hypoxia).

#### Gross morphology

**Figure 2:**
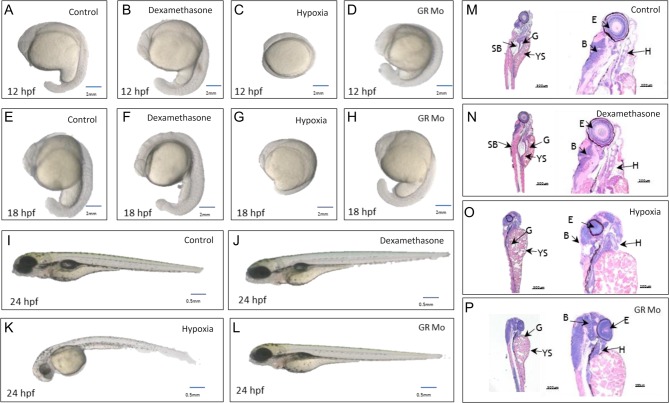
Effects of embryonic treatment on gross morphology of larvae. Images of whole larvae at 12h post fertilisation (hpf) (A, B, C and D), 18hpf (E, F, G and H) and 120hpf (I, J, K and L) show the effects of dexamethasone (B, F and J), hypoxia (C, G and K) and GR morpholino knockdown (GR Mo (D,h and L)) compared with untreated controls (A, E and I). Hypoxic larvae show reduced tail length and larger yolk sac and craniofacial immaturity. By contrast, dexamethasone-treated larvae show advanced eye development, somite patterning and yolk sac puckering. The delay in maturation in GR Mo is less prominent. The swim bladder appears less inflated in larvae with hypoxia and GR Mo treatment. Histological images of H&E-stained sections at two levels of magnification show typical examples of control (M), dexamethasone- (N), hypoxia- (O) and GR Mo-treated larvae at 120hpf (P). Features highlighted are swim bladder (SB), gut (G), yolk sac (YS), eye (E), heart (H) and brain (B). Analyses of images from 20 larvae/group were carried out in consultation with a veterinary pathologist.

All manipulations carried out in this current study were optimised based on dose-ranging/O_2_ saturation studies (Supplementary Fig. 1). None of the treatments used for ongoing experiments impacted significantly on mortality or structural phenotype of larvae (Supplementary Fig. 1). A more subtle delay in development was observed by carefully assessing the gross development in the embryo in reference to an established staging protocol. Histological examination of treated larvae showed no significant differences in the cellular architecture or structure of key non-cardiovascular organs at 120hpf (Kimmel *et al*. 1995) (Fig. 2) with one exception. Both GR Mo- and hypoxia-treated larvae showed reduced frequency of swim bladder inflation at 120hpf (hypoxia: 6.5%±1.8% vs 99.4%±0.2%, *P*<0.001; GR Mo: (25.4%±3.4% vs 99.4%±0.2%, *P*<0.001).

### Larval movement, swimming and behaviour

#### Twitching

**Figure 3:**
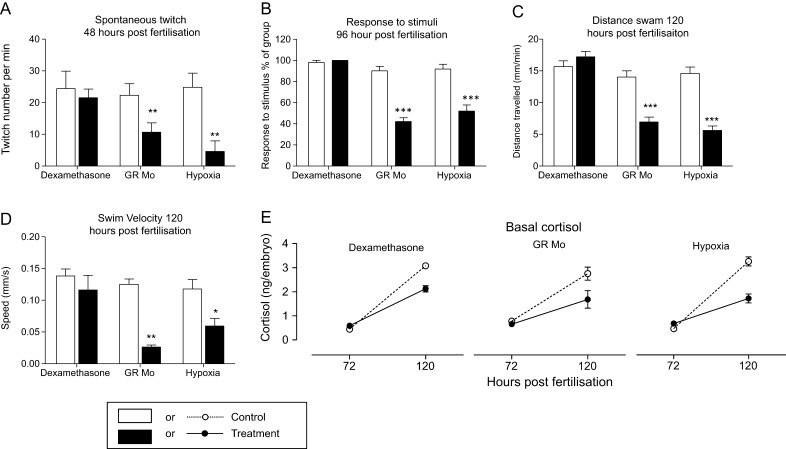
Effects of embryonic treatments on movement, tactile responses, swimming and cortisol levels. Results show the effects of dexamethasone, GR morpholino knockdown (GR Mo) and hypoxia treatment on: (A) spontaneous twitch movement at 48h post fertilisation (hpf (*n*=3 experiments, 20 larvae per group)); (B) tactile responses of 96hpf larvae (*n*=5 experiments, 10 larvae per experiment); (C) Swim distance (mm/min) and (D) velocity during active swimming (mm/s) of 120hpf larvae averaged over a 5min period of observation (*n*=12 larvae); (E) developmental changes in whole larvae cortisol from 72 to 120hpf (*n*=3 experiments, 15 larvae pooled/group). Values are mean±s.e.m. and were analysed by one-way ANOVA and Bonferroni *post hoc* analysis.

The impact of treatments on twitch responses is shown in Fig. 3A. GR Mo and hypoxia showed 60 and 80% fewer twitches, respectively, compared with controls (GR Mo: 10.7%±0.9% vs controls 22.3%±1.1%, *P*<0.01; hypoxia 5.6%±0.8% vs 25%±0.9% twitches per min, *P*<0.001) while dexamethasone appeared to have no impact on this behaviour.

#### Tactile reflexes

Dexamethasone-treated larvae at 96hpf showed no alteration in response to the gentle touch of a forcep tip. Responses of hypoxia and GR Mo larvae were reduced compared with their respective controls (GR Mo: 42%±3.74% vs 90%±4.47% and hypoxia 52%±5.83% vs 91.8%±4.52% both *P*<0.001).

#### Swimming

Swimming activity in larvae at 120hpf was not affected by dexamethasone treatment (Fig. 3C and D). Larvae treated with GR Mo or exposed to hypoxia showed reductions in velocity and in distance travelled compared with their controls (GR Mo *P*<0.01, hypoxia *P*<0.05). Swim behaviour was affected by all treatments. Dexamethasone-treated larvae favoured outer over inner regions of the tank, whereas the opposite pattern was observed in GR Mo and hypoxic larvae.

#### Larval basal cortisol

In line with suggestions that embryonic cortisol is initially derived from maternal sources, there were no differences in cortisol levels for any experimental group at 72hpf compared with controls (all *P* > 0.05; Fig. 3E). However, by 120hpf, larvae treated with dexamethasone, GR Mo or hypoxia all showed reduced cortisol compared with their respective controls (*P*<0.01).

### Larval expression of corticosteroid and growth-related genes

Expression of genes controlling steroid hormone responses and growth showed changes during the time course of development and were altered in response to each of the three experimental treatments (Table 1). Irrespective of treatment, mRNA expression of *mr*, *fkbp5* and *igf* genes were greater at 120 than 72hpf, while *gr*, *hsd2* and *cyp11c1* mRNA levels were unchanged during development. Dexamethasone and hypoxia treatments also differentially affected these genes during the time course of development. At 72hpf, expression of *mr* and *igf* (but not *hsd2*, *gr*, *fkbp5* or *cyp11c1*) were increased by dexamethasone treatment, whereas at 120hpf, *cyp11c1* was downregulated and *hsd2* upregulated. With hypoxia, *igf* expression was lower but only at 72hpf; other genes were unaffected at either 72 or 120hpf. Gene expression patterns were consistently downregulated by GR Mo treatment with *cyp11c1*, *gr* and *igf* reduced at 72 and 120hpf, and *hsd2*, *fkbp5* and *mr* unchanged at these two time points. When gene expression patterns were compared within experimental groups, it was notable in GR Mo-treated larvae that *gr*, *fk506*, *cyp11c1* and *igf* mRNA levels correlated with each other and, in the dexamethasone experiment, *hsd2*, *mr* and *fk506* levels were correlated.

**Table 1:** Developmental- and treatment-specific changes of corticosteroid-related genes in larval fish.

**Relative mRNA abundance** (AU)												
	Hours post fertilisation	*igf*						*fkbp5*					
	Con	Dex	Con Mo	GR Mo	Hyp con	Hyp	Con	Dex	Con Mo	GR Mo	Hyp con	Hyp
72	1.76±0.06	2.34±0.18	1.77±0.03	0.98±0.02**	1.67±0.01	0.87±0.04	6.0±1.1	11.3±1.21*	5.57±1.79	3.50±0.96	5.98±2.6	4.0±1.9
120	2.76±0.30	2.43±0.30	2.67±0.03	1.24±0.5***	2.77±0.07	0.8±0.1	9.0±2.2	14.00±0.50*	9.34±1.9	3.77±0.95*	8.97±1.1	9.50±2.57
	*gr*						*mr*					
	Con	Dex	Con Mo	GR Mo (splice)	Hyp con	Hyp	Con	Dex	Con Mo	GR Mo	Hyp con	Hyp
72	1.04±0.06	1.09±0.01	1.02±0.01	0.41±0.01***	1.00±0.06	0.90±0.04	0.41±0.04	0.74±0.06*	0.39±0.1	0.35±0.08	0.40±0.1	0.33±006
120	0.97±0.03	0.96±0.04	1.04±0.06	0.71±0.03*	1.02±0.1	1.24±0.12	0.76±0.02	1.07±0.06**	0.70±0.1	0.59±0.04	0.69±0.1	0.64±0.2
	*hsd11b2*						*cyp11c1*					
	Con	Dex	Con Mo	GR Mo	Hyp con	Hyp	Con	Dex	Con Mo	GR Mo	Hyp con	Hyp
72	0.91±0.09	1.27±0.01**	0.93±0.03	0.99±0.1	0.99±0.3	0.95±0.04	1.60±0.06	1.80±0.09	1.65±0.01	0.60±0.05	1.7±0.09	1.53±0.01
120	0.76±0.08	1.38±0.09**	0.80±01	1.03±0.05*	0.87±0.12	0.83±0.1	1.79±0.1	1.04±0.04	1.56±0.9	0.70±0.01	1.80±0.04	1.80±0.09

Relative mRNA abundance of genes in larvae treated with (a) dexamethasone, (b) hypoxia and (c) in GR morpholino at 72 and 120hpf. Results were analysed by two-way ANOVA. Effects of time are described in the text. Significant effects of experimental treatment vs respective controls at each time point are *P < 0.05, **P < 0.01 and ***P<0.001 (n= 6–8 groups of 10 fish).

### Adult growth and development

#### Growth

**Figure 4:**
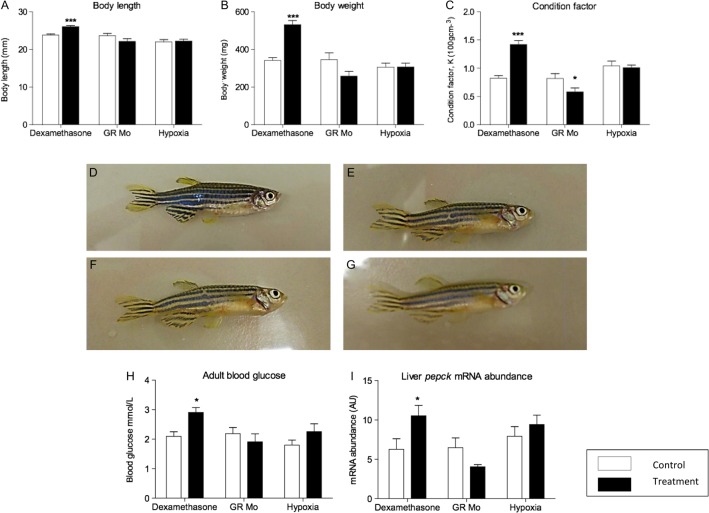
Effect of embryonic treatment on adult body mass, blood glucose and hepatic pepck expression. The effects of embryonic pre-treatment with dexamethasone, hypoxia and GR Mo on body length (A), mass (B) and condition (C) are shown for adult fish at 84dpf. Representative images of adult control (D), dexamethasone (E), hypoxic (F) and GR Mo pre-treated (G) fish show no signs of gross abnormality. Effects of embryonic treatment on adult blood glucose (H) and hepatic *pepck* mRNA expression (I). All data are mean±s.e.m. compared against respective controls by Student’s *t*-test (*P ≤ 0.05, ***P ≤ 0.001). Data for A, B and C are *n*=10 fish, data forh are *n*=8 fish (mean of 2 readings per fish) and *n*=3 (5 pooled livers per sample) for mRNA expression. A full colour version of this figure is available at http://dx.doi.org/10.1530/JOE-15-0376.

Adult fish pre-treated with dexamethasone were longer (Fig. 4A) and heavier (Fig. 4B) than controls resulting in an increase in condition factor value (Fig. 4C). In contrast, the condition factor for GR Mo adults was reduced compared with controls although, separately, reductions in body length and weight were not statistically significant. Hypoxia during early life had no effect on adult growth parameters. As the initial treatment protocols were undertaken on a large heterogeneous group of male and female embryos, the outcome on adult growth could have been influenced by sex ratios. However, there was no difference in male:female ratios in any of the three treatment groups compared with controls (female dexamethasone 52% vs control 58%, *P*=0.66; female GR Mo 53% vs control 60%, *P*=0.53 and female hypoxia 49% vs 59%, *P*=0.79). There were no differences in growth parameters between treated and control fish by gender (data not shown).

#### Gross morphology

Fish were carefully observed throughout development for structural abnormalities, such as tail kinking, craniofacial abnormalities or skin discoloration. There was no excess of abnormal structural features in any of the three treatment groups (Fig. 4G, H, I and J). At 10dpf, fewer hypoxia-treated fish were observed to be free swimming compared with controls (38%±2% vs 100%±0.5%; *P*<0.05) consistent with the findings in the open-field swim assessment (see below). By 20dpf, however, most fish in this group were able to swim freely and observations were comparable to controls (97%±3% vs 98%±0.9%). GR Mo fish, at 10dpf, were similarly affected with fewer fish showing free-swimming behaviour (78%±1.5% vs 100%±0.4%, *P*<0.05) with no differences between treatment groups and controls by 20dpf (98%±1.3% vs 97%±1.0%). Fish treated with dexamethasone as embryos had comparable free-swimming behaviour as controls at both 10 and 20dpf (dexamethasone 98%±1.2% vs controls 99%±0.8% and dexamethasone 99%±0.9% vs control 100%±1.5%, respectively).

#### Adult metabolic alterations

As markers of glyco­lytic metabolism, plasma glucose levels and hepatic expression of phosphoenolpyruvate carboxykinase (*pepck)* gene were measured in adult fish derived from each of the three larval treatment groups (Fig. 4K and L). In adult zebrafish, hepatic *pepck* was increased in the dexamethasone-treated group, decreased in GR Mo and unchanged in the hypoxia group. However, only adults in the dexamethasone group showed changes in blood glucose (Fig. 4K and L). Expression of *mr* and *gr* genes following larval treatment in adult liver, kidney, brain and skeletal muscle tissue are shown in Table 2. Dexamethasone treatment increased the expression of gr in kidney only. Hypoxia decreased *gr* in muscle while GR Mo treatment reduced gr in the liver. None of the treatments affected the expression of *mr* in any of the tissues studied.

**Table 2:** Effects of embryonic treatment on tissue-specific expression of corticosteroid receptors in adult fish.

**Relative mRNA abundance** (AU)												
	*gr*						*mr*					
Tissue	Con	Dex	Con Mo	GR Mo	Hyp con	Hyp	Con	Dex	Con Mo	GR Mo	Hyp con	Hyp
Liver	0.34±0.05	0.47±0.03	0.37±0.03	0.21±0.02*	0.42±0.04	0.31±0.34	0.62±0.02	0.68±0.03	0.71±0.07	0.62±0.08	0.70±0.05	0.59±0.09
Kidney	1.17±0.17	2.03±0.07**	1.20±0.26	1.45±0.09	1.20±0.24	1.30±0.14	1.20±0.09	1.12±0.05	1.32±0.06	1.28±0.05	1.18±0.08	1.21±0.04
Brain	6.35±0.59	7.23±0.85	7.01±0.65	6.74±0.14	5.98±0.65	4.56±0.34	10.05±0.60	11.7±0.06	9.35±0.31	10.3±0.4	9.98±1.21	10.01±0.7
Muscle	2.48±0.09	2.34±0.04	2.01±0.23	2.10±0.12	3.00±0.09	2.13±0.10	0.85±0.06	0.89±0.02	0.91±0.02	0.87±0.03*	0.67±0.06	0.71±0.05

Relative mRNA abundance of *mr* and *gr* in adult fish tissues after embryonic/larval treatment. Results were analysed by two-way ANOVA. Significant effects of pre-treatment indicated by *P<0.05 and **P<0.001.

### Adult swim activity and behaviour

#### Forced swim/dive assay

**Figure 5:**
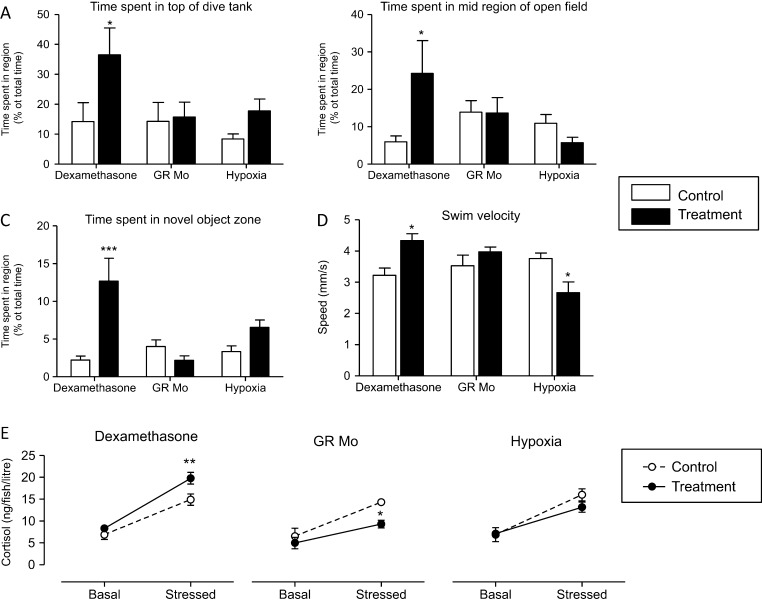
Effect of embryonic treatment on adult behaviour and stress-induced changes in cortisol release. The effects of embryonic pre-treatment with dexamethasone, hypoxia and GR Mo on: (A) dive responses; (B) behaviour in an open-field test; (C) avoidance behaviour in response to a novel object and (D) swim velocity were analysed in adult fish. Values (mean±s.e.m., *n*=10) were analysed by one-way ANOVA and Bonferroni *post hoc* analysis, *P≤0.05, ***P≤0.001). Basal and stress-induced cortisol levels in swim water (E) from *n*=3 experiments (5 fish per tank per group) were analysed by one-way ANOVA and Bonferroni *post hoc* analysis, **P≤0.01.

The time spent by fish in upper and lower regions of a novel tank is shown in Fig. 5A. Control fish from all treatment groups spent approximately 85% of the time in the lower region of the tank. This behaviour was not affected in either the GR Mo or hypoxia adults. However, dexamethasone adults spent longer exploring the upper region of the tank compared with controls (36.5%±8.9% vs 14.2%±%6.4%, *P*=0.05) and correspondingly less time in the lower region (53.3%±8.9% vs controls 89.2%±4.2%, *P*=0.05).

#### Open-field assay

In open-field tests (Fig. 5B), the percentage of time spent in the central zone by fish pre-treated with dexamethasone was less than controls, whereas GR Mo and hypoxia fish spent less time in the centre than their respective controls. When a novel object was placed at the centre of the tank, dexamethasone adults spent five times longer in the novel object zone than their controls (Fig. 5C), but neither hypoxia nor GR Mo adults spent significantly more time in the vicinity of the novel object.

#### Swim velocity

Swim velocity (the speed at which the fish travelled whilst exploring the tank) was reduced in adults pre-treated with hypoxia and increased in adults pre-treated with dexamethasone (Fig. 5D). GR Mo fish swam at the same speed as controls.

#### HPI activity

Cortisol levels in the water of a 5L tank containing 5 adult fish for 24h periods were analysed without (basal) and with stress (Fig. 5E). Basal values were not significantly affected by (embryonic treatment with) dexamethasone, hypoxia or GR Mo when compared with respective controls. Netting stress caused cortisol levels to increase in all groups including controls for each treatment by at least 200%. Stress-induced cortisol levels were higher in adult fish, derived from dexamethasone-treated embryos, compared with their adult controls. In contrast, stress-induced cortisol levels were reduced in GR Mo and hypoxia groups compared with controls.

## Discussion

The effects of early-life pharmacological, genetic and physiological manipulations on the short- and long-term growth and development of larval zebrafish have been investigated in three experimental models: (i) excess GC activity by treatment with the synthetic hormone dexamethasone; (ii) reduced GC activity by injection with an antisense GR Mo; and (iii) exposure to hypoxia, a physiological stressor known to affect behaviour, gross morphology and locomotor activity (Ton *et al*. 2003, Shang & Wu 2004, Shang *et al*. 2006, Widmer *et al*. 2006, Marks *et al*. 2012). These experiments tested the hypothesis that transient GC manipulations during early life would have direct effects on morphology, endocrine responses and behaviour at or close to the end of the treatment phase (120hpf) and that this would lead to biological consequences in adults akin to the programming effects of GCs previously reported in mammalian models. Novel aspects addressed in these experiments are the impact of early-life hypoxia and GC manipulation in the absence of placental or maternal influences.

### Larval effects of glucocorticoid manipulation

#### Glucocorticoids play a key role in development, growth and hatching

Previous studies have shown that although the source of corticosteroids in zebrafish embryos over the first 24–36hpf is exclusively maternal, thereafter steroidogenesis slowly becomes autonomous (Alsop & Vijayan 2008, Tokarz et al. 2013a, b, Wilson *et al*. 2013). However, since *gr* expression has been identified from at least 24hpf, we can assume that GCs from whatever source have the potential to influence development at every stage of early life. This is confirmed in our work in several ways. Hatching, when larvae emerge from the chorion, normally takes place at or around 48hpf at a time when the source of steroid is predominantly maternal. Augmenting endogenous steroid by treating eggs with dexamethasone caused the average time of hatching to advance by 8h, whereas reducing GC action, by injection with a GR Mo, delayed hatching by 5–8h as similarly reported by Nesan and Vijayan (2013) and previously by ourselves (Wilson *et al*. 2013). Therefore, hatching appears to be modulated by GCs at a stage of development when endogenous hormone is exclusively maternal. Similar effects were noted for head–trunk angle, with an acceleration observed in dexamethasone treatment. Delays in GR Mo were partially rescued with the introduction of *gr* mRNA rescue (Supplementary Fig. 2). These data suggest the importance of GCs at critical stages of embryonic development and are supported by previous studies in zebrafish and other fish species. In medaka, hatching was advanced and delayed by cortisol treatment and GR knockdown, respectively (Trayer *et al*. 2013). In contrast, treatment of Rainbow trout oocytes with cortisol increased zygote activation and cytogenesis but did not affect percentages of egg fertilisation or hatching (Kusakabe *et al*. 2002). Similarly, when spawning cod were treated with cortisol, increased levels of egg cortisol were associated with greater expression of genes involved in cytogenesis although again no significant effect on hatching was observed (Kleppe *et al*. 2013). Others have noted that hatching may be advanced by GCs but that the percentages of eggs that hatch are reduced (Kleppe *et al*. 2013, Trayer *et al*. 2013).

The early effects of GCs extend beyond the period of hatching and are reflected in a number of parameters such as head–trunk angle, tactile responses, somatic growth and locomotion. Although opposing effects of dexamethasone and GR Mo on *fkbp* gene expression are evidence of GC involvement, it is significant that temporal phenotypic difference were observed generally as a delay in GR Mo larvae rather than advancement with dexamethasone. Previous studies have demonstrated that GR Mo treatment has dose-dependent effects on mesodermal structures (Pikulkaew *et al*. 2011, Nesan & Vijayan 2013). In this study, we titrated the dose of GR Mo to avoid non-specific abnormalities such as pericardial oedema and craniofacial abnormalities and confirmed specificity by partial rescue with capped *gr* mRNA (Supplementary Fig. 1).

Growth effects could be secondary to other phenotypes. Locomotor activity, which is reduced in GR Mo, has been shown to promote skeletal muscle growth in adults (Palstra *et al*. 2014). Similarly, a delay in swim bladder inflation in GR Mo could indicate changes in hydrostatic function that indirectly affect metabolism. Understanding the molecular mechanisms underlying GC effects on growth is complicated by the diverse nature of GR-regulated processes. For example, it has been shown that pancreatic beta cell proliferation is stimulated by GCs (Tsuji *et al*. 2014) and, since the insulin signalling system is a key determinant of somatic growth, the effects of GR Mo could be secondary to impaired insulin secretion. It has also been shown that at 72hpf, GCs regulate ion transporters which affect survival by maintaining water and electrolyte homeostasis (Kumai *et al*. 2012). Another critical factor regulating growth before and after hatching is the *igf* system. We and others have implicated *igf* in the development of the cardiovascular and central nervous system, and when *igf* responses are impaired, growth is reduced (Hartnett *et al*. 2010). It is notable that *igf* gene expression is reduced in GR Mo at both 72 and 120hpf.

Developmental delays similar to those of GR Mo were seen with hypoxia, a physiological stressor that could reflect natural adverse environmental conditions such as stagnant water. Again, the severity of treatment in the current study was titrated to avoid gross effects on survival. The main embryonic effects of hypoxia appears to be a delay in hatching and is accompanied by changes in other markers including head–trunk angle, inflation of the swim bladder, development of tactile responses, locomotor activity and growth rates. Like GR Mo and dexamethasone, the effects of hypoxia are associated with changes in *igf* expression but unlike GR Mo there were no changes in other genes determining cortisol signalling and responses. Moreover, the initial delay was observed during a period before embryonic HPI function is fully functional. This would suggest that reduced oxygen levels in the bathing water result in a slowing of metabolic processes that underlie development without, at least initially, affecting GC activity. This is perhaps not surprising since the process of hatching has been associated with a 10-fold increase in oxygen utilisation (Barrionuevo & Burggren 1999). It is also significant that *igf* signalling is known to be required for catch-up growth following a period of severe hypoxia at 24hpf (Kamei *et al*. 2011). In other fish species, hypoxia has also been shown to delay hatching (Jones & Reynolds 1999, Kamei *et al*. 2011, ­Robertson *et al*. 2014).

Although there are differences in molecular responses among dexamethasone, GR Mo and hypoxic larvae, it is notable that basal cortisol levels were reduced by all treatments. Here, we discuss several factors that might account for these responses: (i) negative feedback control of cortisol secretion; (ii) the maturation of the HPI axis: (iii) the requirement of oxygen for steroidogenesis; (iv) the clearance of cortisol.

Previously, we showed that acute treatment with dexamethasone at later stages of larval development reduced cortisol levels consistent with ideas of fast-­negative feedback control of the HPI axis. However, To and coworkers showed that chronic dexamethasone treatment caused a more profound suppression of the HPI axis (To *et al*. 2007). Pituitary *pomc* and interrenal *cyp11a1* expression were reduced with a more potent effect on *cyp11a1* at 120 than 72hpf. *cyp11a1* encodes cholesterol side-chain cleavage, the first rate-limiting step in steroidogenesis. Although cortisol was not measured by To and coworkers, their results are consistent with the present findings where dexamethasone-induced cortisol reductions were greater at 120hpf, and *cyp11c1*, which encodes the final enzyme in the cortisol pathway, was significantly lower at 120 but not 72hpf.

Recently, Parajes and coworkers demonstrated that the expression of the paralog Cyp11a2, which is not easily distinguished from Cyp11a1, determines cortisol stress synthesis in larval zebrafish (Parajes *et al*. 2013). Similarly, we have noted that cortisol levels are reduced at later stages of development by pharmacologic inhibition of Cyp11c1 activity (Wilson *et al*. 2013) and in Cyp11c1 morphants. In this study, it seems likely that reduced *cyp11c1* levels cause reductions in cortisol levels in GR Mo which limits GC activity. It is significant that *gr* and *cyp11c1* mRNA expression patterns across both 72 and 120hpf time points are closely correlated with each other and with *fkbp5* – an established GC response gene. In some respects, this pattern is paradoxical, in that GR mediates negative feedback control of cortisol on Acth secretion. Indeed, GR-knockout mice (GR^-/-^) have high plasma levels of corticosteroid hormones and markedly hypertrophied adrenocortical tissue (Cole *et al*. 1995). A possible explanation for this paradox in GR Mo might involve a fish-specific process of Acth stimulation of cortisol synthesis. Although Mcr2 (melanocortin receptor type 2) is recognised as the canonical receptor for Acth regulation of cortisol in teleost fish, Mcr4, which is associated with growth in embryonic/larval zebrafish, is expressed in head kidney (the locus of interrenal cells) and has been shown to be responsive to Acth (Josep Agulleiro *et al*. 2013) when associated with melanocortin receptor accessory proteins (also expressed in head kidney). The activity of Mcr4 in larval fish is inhibited by Mrap2a (a larval MRAP paralog which inhibits Mcr4 actions) (Sebag *et al*. 2013). As GCs have been shown to downregulate *mrap2a* expression (Josep Agulleiro *et al*. 2013), it may be that inhibitory effects of Mrap2a on Mcr4 are enhanced in GR Mo leading to decreased Acth responsiveness. However, whether Mcr4 contributes to the control of cortisol synthesis in zebrafish larvae has yet to be established.

Hypoxic fish also have reduced cortisol levels and *igf* expression but showed no effect on *cyp11c1* expression or other markers of corticosteroid signalling. However, cortisol biosynthesis is dependent on oxygen availability: mammalian Cyp11a and Cyp11b genes encode mitochondrial steroidogenic enzymes which require oxygen as a cosubstrate. Previous studies with mammalian cells and isolated mitochondria have shown that the availability of oxygen, particularly at early stages of development, markedly affects cholesterol chain cleavage (CYP11A1) and 11β-hydroxylase (CYP11B1) activities (Stevens *et al*. 1984, Bruder *et al*. 2002). It follows that, without changing steroidogenic enzyme expression, hypoxia in zebrafish could impair interrenal steroidogenesis.

A final factor to consider in the maintenance of larval cortisol levels is inactivation by Hsd11b2. Only dexamethasone treatment increased *hsd11β2* gene expression, and since enzyme activity is thought to regulate hormone availability in specific target tissues, the contribution this makes to whole-body cortisol turnover may be minor. It is interesting that in mammals, HSD11Β2 is thought to facilitate aldosterone binding to mineralocorticoid receptor (MR). Zebrafish lack aldosterone but changes in *hsd11b2* expression parallel to those of *mr* suggesting that these may compensate for reduced endogenous ligand (cortisol) (Tokarz et al. 2013a, b).

A further consideration of larval development is the effect of treatments on behaviour. Thigmotaxis or ‘wall hugging’ is a natural anxiogenic response to predators which is normally seen from 96hpf (Colwill & Creton 2011) and may, therefore, be subject to the developmental delay. It is significant that larvae treated with dexamethasone spent more time in outer zones than controls, whereas GR Mo and hypoxia larva spent less time. Arguably, these responses could be a direct function of GC activity since, in adult fish, drug and genetic manipulations which promote anxiogenic and anxiolytic responses are associated with increased and reduced GC exposure, respectively (reviewed by Colwill & Creton 2011).

### Adult programming

In addition to contemporaneous effects of these treatments in larval fish, we present evidence that early-life treatments have consequential effects on a number of adult phenotypes including behaviour, metabolism and the HPI axis. This programming phenomenon has been widely studied in mammals (Cottrell & Ozanne 2008, Cederroth & Nef 2009, Eriksen *et al*. 2011) but is not without precedent in fish (Fernandino *et al*. 2012, Oswald *et al*. 2012). For example, hypoxia and the increasing cortisol content of fish eggs have been shown to affect the male:female ratio of survivors and as well as weight gain and various behavioural traits (Shang *et al*. 2006, Oswald *et al*. 2012). In this study, none of the early-life treatments affected the subsequent numbers of male and female adult fish, but it was notable that condition factor was raised and lowered in dexamethasone and GR Mo groups, respectively. In rodents, early-life exposure to GCs causes intrauterine growth retardation followed by a period of post-natal catch-up growth and is associated in adults with tissue-specific alterations to *Gr* mRNA expression, including t is associated in adults with tissue-specific alterations he liver, leading to hepatic expression of the gluconeogenic enzyme *pepck* and hyperglycaemia. Although blood glucose levels and *pepck* gene expression were increased in adult fish derived from larvae treated with dexamethasone, these changes were not entirely consistent with the observed changes in expression of *gr* or *mr* mRNA in liver, muscle or brain. A more likely rationale for adult metabolic programming effects of larval treatment are the changes in cortisol levels observed in stressed adult fish. The underlying cause of programmed effects on HPI activity require further investigation but in theory could stem from early embryonic influences. In Atlantic salmon and trout, increased egg cortisol content has been linked to aggressive behaviour in juvenile fish (Sloman 2010). Arguably, this trait might influence feeding activity with effects on body mass. It is debatable that aggression equates with boldness, but it is significant that by three test criteria, dexamethasone pre-treated adults were bolder and GR Mo were more reserved. These observations contrast with those of Baiamonte and coworkers (2016) who suggested that decreased thigmotaxis in adult zebrafish after embryonic exposure to ethanol was associated with transient decreased HPI activity and with those of Kohr and coworkers who noted that dexamethasone pre-treatment caused adult fish to remain at the bottom of the test tank rather than to explore upper zones (Khor *et al*. 2013). It should be noted, however, that the schedules of steroid treatment were different in our experiments and as highlighted by Bailey and coworkers (2015) exposure windows may determine cellular and molecular mechanisms to functional neurobehavioral effects. Kohr and coworkers started dexamethasone treatment at 96hpf and finished at 120hpf, whereas treatments in the present experiment were continuous from 0–120hpf and included embryonic stages, which are well recognised to be critically important in development. Indeed, this early stage of development has been proposed as a period of high GC sensitivity for the induction of genes involved in metabolism (Chatzopoulou *et al*. 2015) and as a window for the initiation of long-term programming of behaviour (Sloman 2010). In the ethanol paradigm of Baiamonte and coworkers (2015), treatment was maintained for longer period (9dpf). Reduced thigmotaxis was associated with diminished transient cortisol responses to stress, but other known behavioural effects of ethanol were not investigated. We suggest, bearing in mind that the behavioural effects of GCs in larvae and adults in the present experiment are very different, that steroid treatment evokes responses which are dependent on whether fish have hatched or not.

## Conclusion

Manipulating GC exposure embryonic and larval fish by pharmacological, genetic and environmental interventions affects the timing of development and the maturation of the HPI axis. Early-life variations in GC activity are associated with subsequent behavioural and metabolic changes in adults. These effects may differ depending on whether treatment is administered before or after autonomous steroidogenesis when larvae are responsive to stress. Further studies of the temporal effects of steroids in zebrafish may help unravel the underlying cellular and molecular mechanisms involved in programming of adult disease.

## Supplementary data

This is linked to the online version of the paper at http://dx.doi.org/10.1530/JOE-15-0376.

## Declaration of interest

The authors declare that there is no conflict of interest that could be perceived as prejudicing the impartiality of the research reported. All authors takes responsibility for all aspects of the reliability and freedom from bias of the data presented and their discussed interpretation.

## Funding

This study was funded by a British Heart Foundation PhD Fellowship award (KW) grant number RE/09/053. The work was also supported by the British Heart Foundation Centre of Research Excellence award (CoRE) grant number RE/08/001/23904.
